# Pancreaticobiliary Cancers and Aeromonas Isolates Carrying Type Ⅲ Secretion System Genes *ascF-ascG* Are Associated With Increased Mortality: An Analysis of 164 Aeromonas Infection Episodes in Southern Taiwan

**DOI:** 10.3389/fcimb.2021.749269

**Published:** 2021-10-19

**Authors:** Ying-Wen Chen, Shu-Li Su, Chia-Wen Li, Chin-Shiang Tsai, Ching-Lung Lo, Ling-Shan Syue, Min-Chi Li, Ching-Chi Lee, Nan-Yao Lee, Wen-Chien Ko, Po-Lin Chen

**Affiliations:** ^1^ Department of Internal Medicine, National Cheng Kung University Hospital, College of Medicine, National Cheng Kung University, Tainan, Taiwan; ^2^ Diagnostic Microbiology and Antimicrobial Resistance Laboratory, National Cheng Kung University Hospital, College of Medicine, National Cheng Kung University, Tainan, Taiwan; ^3^ Infection Control Center, National Cheng Kung University Hospital, College of Medicine, National Cheng Kung University, Tainan, Taiwan; ^4^ Department of Medicine, College of Medicine, National Cheng Kung University, Tainan, Taiwan; ^5^ Clinical Medicine Research Center, National Cheng Kung University Hospital, College of Medicine, National Cheng Kung University, Tainan, Taiwan; ^6^ Department of Microbiology and Immunology, College of Medicine, National Cheng Kung University, Tainan, Taiwan

**Keywords:** *Aeromonas*, identification, *rpoD* sequencing, ascF-ascG, type 3 secretion system, antimicrobial resistance, virulence, pancreaticobiliary cancers

## Abstract

This prospective study aimed to investigate the clinical and microbiological characteristics of different *Aeromonas* species. Clinical isolates of *Aeromonas* species between 2016 to 2018 were collected in a university hospital in southern Taiwan. The species was determined by *rpoD* or *gyrB* sequencing. A total of 222 *Aeromonas* isolates from 160 patients in 164 episodes were identified. The crude in-hospital mortality was 17.2%. The most frequently isolated species was *Aeromonas veronii* (30.6%), followed by *A. caviae* (24.8%), *A. hydrophila* (23%), and *A. dhakensis* (16.7%). The major clinical manifestations were primary bacteremia (31.1%), skin and soft tissue infection (22.6%), and biliary tract infection (18.3%). The most common underlying diseases were malignancy (45.1%), diabetes mellitus (27.4%), and liver cirrhosis or chronic hepatitis (26.2%). *A. hydrophila* and *A. dhakensis* predominated in the skin and soft tissue infection (p<0.0001), whereas *A. vernoii* and *A. caviae* prevailed in primary bacteremia and biliary tract infections (p=0.012). Pneumonia, malignancy, and *ascF-ascG* genotype were independent factors associated with mortality. Ertapenem susceptibility was decreased in *A. sobria* (42.9%), *A. veronii* (66.7%), *A. dhakensis* (73%), and *A. hydrophila* (84.3%). Cefotaxime resistance was found in 30.9% of *A. caviae* and 18.9% of *A. dhakensis* isolates, much more prevalent than the other species. The metallo-β-lactamase *bla_CphA_* was almost invariably present in *A. dhakensis*, *A. hydrophila*, and *A. veronii* (100%, 100% and 89.9%, respectively). Amp-C β-lactamases such as *bla_MOX_* and *bla_AQU-1_* were identified in all *A. caviae* and 91.9% of *A. dhakensis* isolates. Cefepime, fluoroquinolones and tigecycline showed good *in vitro* activity against aeromonads.

## Introduction

The *Aeromonas* species are Gram-negative, rod-shaped bacteria that inhabited soil and aquatic environment ubiquitously, from fresh and brackish water, seawater, groundwater, sewage to drinking water. In addition, they were also found in ﻿fish and seafood, dairy, meats, and vegetables ﻿intended for human consumption (Fernández-Bravo and Figueras, 2020). They cause a wide spectrum of diseases in humans, notably acute gastroenteritis, septicemia, and soft tissue infections, as well as hepatobiliary tract infections, peritonitis, respiratory tract infections, urogenital tract infections, indwelling-device related infections, ocular infections, meningitis, and hemolytic uremic syndrome (Janda and Abbott, 2010).

The pathogenicity of aeromonads is complex owing to their multiple virulence factors acting collectively or separately, including structural components like flagella, adhesins, lipopolysaccharide and capsule, extracellular enzymes like lipases, proteases, elastases, and hemolytic enzymes that cause cell and tissue damage, enterotoxins that induce diarrhea, and most importantly the type III secretion system (T3SS) that injects toxins directly into host cells. The T3SS is composed of thorn-shaped or syringe structure, effector proteins that are injected, and chaperones that assist and protect structural and effector proteins during transport (Tomás, 2012; Rasmussen-Ivey et al., 2016; Gonçalves Pessoa et al., 2019). AscV serves as an indicator for the presence of the type III secretion machinery. AscF-AscG serves as translocation apparatus (Vilches et al., 2004; Chacón et al., 2004; Burr and Frey, 2007). AexT is an effector protein possessing ADP-ribosyltransferase and GTPase acting protein activities and is homologous to the *Pseudomonas aeruginosa* effector ExoT/ExoS (Braun et al., 2002; Tomás, 2012). The cytotoxic enterotoxin Act provokes the degeneration of intestinal epithelium and leads to bloody diarrhea, while the cytotonic enterotoxins, including heat-stable type Ast and heat-labile type Alt, cause non-bloody diarrhea (Gonçalves Pessoa et al., 2019).

Southern Taiwan locates in a subtropical area and is an *Aeromonas*-prevalent region, with an incidence of *Aeromonas* bacteremia of 76 per million inhabitants per year, much higher than that in western countries with an annual incidence of merely up to 1.5 per million (Wu et al., 2014). Historically, *A. hydrophila* had been the most common species isolated in bacteremia in Southern Taiwan (Ko and Chuang, 1995; Ko et al., 2000; Tang et al., 2014), but recent advances in molecular studies based on 16s RNA (Martínez-Murcia et al., 1992), housekeeping genes (Yáñez et al., 2003; Soler et al., 2004; Martínez-Murcia et al., 2011), and genome sequencing (Colston et al., 2014), had led to ﻿the reclassification of aeromonads. As a result, the reported prevalence of the most predominant clinical species of *Aeromonas* has changed over the years, with most (﻿﻿96.5%) of the aeromonads associated with clinical cases identified as *A. caviae* (29.9%), *A. dhakensis* (26.3%), *A. veronii* (24.8%), and *A. hydrophila* (15.5%) (Fernández-Bravo and Figueras, 2020). ﻿Besides, concordance was low between phylogenetic identification and the commercial identification systems, with incorrect identification at species level (Lamy et al., 2010). ﻿For example, it could be difficult to separate *A. veronii* biovar *sobria* from *A. hydrophila* using conventional biochemical tests. *A. veronii* biovar *sobria* shares common phenotypes with *A. sobria sensu stricto* ﻿and was often reported mistakenly as *A. sobria* (Janda and Abbott, 2010). *A. dhakensis* was mistaken as *A. hydrophila* for decades and is often misidentified as *A. hydrophila*, *A. veronii*, or *A. caviae* by commercial phenotypic tests (Chen et al., 2016). Since 16s RNA is highly conserved in aeromonads, housekeeping genes like *gyrB* (subunit B of DNA gyrase) and *rpoD* (sigma factor S70) offer less mean sequence similarity values and hence higher resolution than the 16s RNA gene (Yáñez et al., 2003; Soler et al., 2004; Martínez-Murcia et al., 2011).

In this prospective study, we investigated patients with clinical isolates of *Aeromonas* species determined by DNA sequence matching of *rpoD* or *gyrB* between 2016 to 2018 in ﻿a medical center in Southern Taiwan. The demographic factors, clinical outcome, drugs susceptibility of *Aeromonas* isolates, and the prevalence of genes responsible for drug resistance and virulence were analyzed. The study aimed to provide a better understanding of the association between clinical spectrum and different *Aeromonas* species determined by molecular typing.

## Methods

### Patients

*Aeromonas* isolates in National Cheng Kung University Hospital, a university-affiliated medical center with approximately 1200 beds located in Tainan, Taiwan, were collected from January 2016 to December 2018. The study was ethically approved by The Institutional Review Board of National Cheng Kung University Hospital (IRB no. A-ER-104-352). Medical chart records were reviewed retrospectively, and information collected included underlying diseases, sites from which specimens were obtained for culture, infectious diseases caused by Aeromonas species, and clinical outcomes. The requirement for informed consent was waived by the Institution Review Board.

### Species Identification

A total of 222 isolates were available for analysis and stored at -70°C until use. ﻿The *Aeromonas* isolates were identified by the MALDI-TOF MS V2.0 (bioMérieux, Marcy-l’Étoile, France), and ﻿species identification of each *Aeromonas* isolates was determined based on the partial sequences of *rpoD* ﻿(and gyrB, if necessary) (Yáñez et al., 2003; Soler et al., 2004). The sequences amplified were compared with reference sequences from the GenBank database using BLAST (http://www.ncbi.nlm.nih.gov/BLAST/). Isolates with a dissimilarity value of ≤1% were considered the same species.

### Detection of Resistance Genes and Virulence Factors

Genes contributing to antibiotic resistance and virulence were detected by polymerase chain reaction (PCR) using previously described primers. Resistance genes included AmpC β-lactamases *bla_AQU-1_* (Wu et al., 2013) and *bla_MOX-like_* (Wu et al., 2015), metallo-β-lactamases (MBL) *bla_CphA_* (Wu et al., 2012), *bla_KPC_*, *bla_IMP_*, *bla_VIM_*, *bla_NDM_*, *bla_OXA-23-like_* and *bla_OXA-48-like_*, and extended-spectrum β-lactamases *bla_TEM_*, *bla_PER_*, *bla_SHV_*, and *bla_CTX-M_* (Wu et al., 2011). Virulence factors included the polar flagellum (*fla*), collagenase (*col*), lipase (*lip*), elastase (*ela*), aerolysin (*aerA*), hemolysin (*hlyA*), heat-stable enterotoxin (*ast*), heat-labile enterotoxin (*alt*), cytotoxic enterotoxin (*act*), and three components of T3SS, *ascV*, *ascF-ascG*, and *aexT*.

### Antimicrobial Drug Susceptibility Testing

The antimicrobial drug susceptibility testing ﻿was determined by the disk diffusion test and interpreted following the Clinical and Laboratory Standards Institute (CLSI) recommendations for *A. hydrophila* complex (Clinical and Laboratory Standards Institute, 2016). ﻿The criteria for tigecycline susceptibility followed the U. S. Food and Drug Administration criteria for *Enterobacteriaceae*.

### Statistical Analysis

Continuous variables are expressed as mean ± standard deviation (S.D.) and compared using the Wilcoxon Rank Sum test or the Student’s independent t-test, as appropriate. Categorical variables were compared using the Chi-square test or Fisher’s exact test ﻿if the expected counts were less than five. A p-value < 0.05 was considered statistically significant. Those variables with a P-value < 0.05 in the univariate analyses were put into a multivariate logistic regression model to adjust for confounding. Statistical analyses were conducted using the statistical package SPSS for Windows (version 22.0, SPSS, Chicago, IL, USA).

## Results

### Patient Characteristics

**Figure 1:**
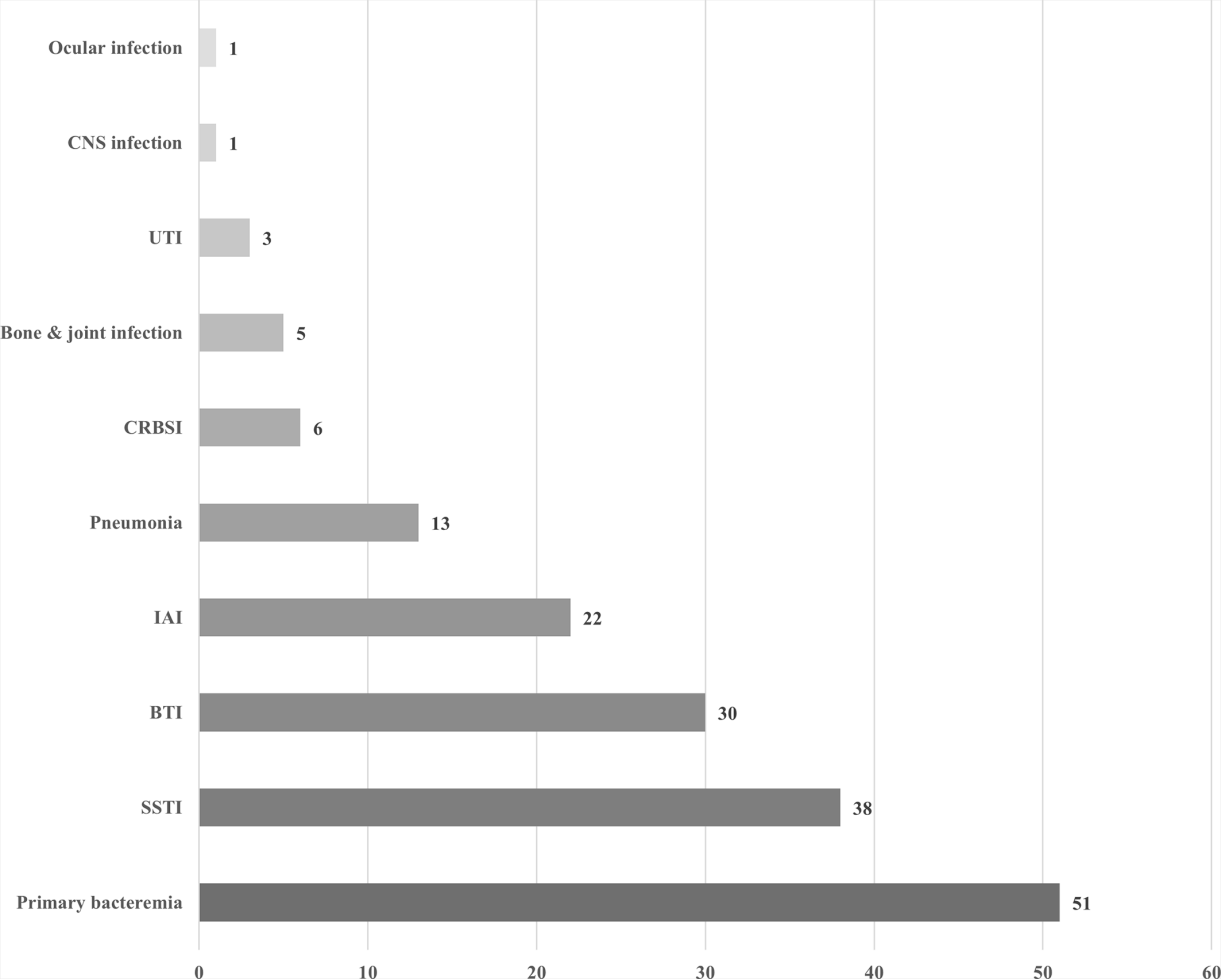
Clinical manifestations of 164 episodes of *Aeromonas* infection. UTI, urinary tract infection; CRBSI, catheter-related bloodstream infection; IAI, intra-abdominal infection; BTI, biliary tract infection; SSTI, skin and soft tissue infection.

During the study period, a total of 222 *Aeromonas* isolates were obtained from 160 patients. Four patients had recurrent episodes of *Aeromonas* infection at least 180 days apart within the study period, yielding a total of 164 episodes. The demographic data and clinical characteristics of the patients are ﻿summarized in **Table 1**. The mean age was 62.9 (S.D. 16.8) years, ranging from 4 months to 93 years. Male patients outnumbered female patients (109/160, 68.1%). The major underlying diseases were active malignancy (72/160, 45.0%), followed by diabetes mellitus (44/160, 27.5%), liver cirrhosis or chronic hepatitis (40/160, 25.0%), and chronic kidney diseases including those receiving renal replacement therapy (29/160, 18.1%). The most common type of cancer in patients with active malignancies was hepatocellular carcinoma (14/72, 19.4%), followed by pancreatic cancer (11/72, 15.3%). Most of the patients in these episodes (145/164, 88.4%) were hospitalized. Ninety-six (58.5%) of the episodes were polymicrobial infections mixed with other bacteria. Seventy-one (43.3%) of the episodes presented with bacteremia. The crude in-hospital mortality was 17.2% (28/163, one missing due to transference to another hospital). As shown in **Figure 1**, the most common clinical manifestations were primary bacteremia (51/164, 31.1%), skin and soft tissue infection (SSTI, 37/164, 22.6%), and biliary tract infection (BTI, 30/164, 18.3%). Biliary tract infection was associated with biliary stones (p=0.001) and pancreatobiliary cancers (including pancreatic cancer, cholangiocarcinoma, and ampullary cancer, p<0.0001), but not with liver cirrhosis/chronic hepatitis (p=0.17) or hepatocellular carcinoma (p=0.72).

**Table 1:** Demographics of 160 patients with clinical *Aeromonas* infections.

Characteristics	No. (%) of Patients
**Age, yr (mean ± standard deviation)**	62.9 ± 16.8
**≥65**	80 (50.0)
**Sex, female**	51 (33.1)
**Underlying disease**	
**Active malignancy**	72 (45.0)
**Hepatocellular carcinoma**	14/72 (19.4)
**Pancreatic cancer**	11/72 (15.3)
**Bile duct cancers**	7/72 (9.7)
**Other gastrointestinal tract cancers**	16/72 (22.2)
**Hematologic dyscrasias**	13/72 (18.1)
**Others**	10/72 (13.9)
**Diabetes mellitus**	44 (27.5)
**Liver cirrhosis/chronic hepatitis**	40 (25.0)
**Chronic kidney disease**	29 (18.1)
**Biliary stone**	17 (10.6)
**Cerebrovascular accident**	9 (5.6)
**Autoimmune disease**	3 (1.9)

### Comparison of the *rpoD* Sequencing and the MALDI-TOF MS System for Identification of *Aeromonas* Species

As shown in **Table 2**, the most common species isolated was *Aeromonas veronii* (69/222, 31.1%), followed by *A. caviae* (55/222, 24.8%), *A. hydrophila* (51/222, 23.0%), and *A. dhakensis* (37/222, 16.7%). The MALDI-TOF MS system correctly identified all isolates at the same genus level with molecular methods, but only 48.2% (107/222) achieved at the same species level. Most of the *A. caviae* (48/55, 87.3%) and *A. hydrophila* (45/51, 88.2%) isolates were identified as *A. hydrophila*/*caviae* using MALDI-TOF MS system. The *A. veronii* isolates had a mere 2.9% concordance at the species level between molecular typing and MALDI-TOF MS, and nearly half of them were as *A. sobria*. The MALDI-TOF MS 2.0 version system is unable to identify *A. dhakensis* due to no corresponding data in the database.

**Table 2:** Comparison of *rpoD* sequencing with MALDI-TOF MS (V2.0).

*rpoD* Sequencing	MALDI-TOF MS	No.	%
*A. dhakensis*, N=37	*A. hydrophila/caviae*	32	86.5%
	*Aeromonas* spp.	3	8.1%
	*A. hydrophila*	2	5.4%
*A. hydrophila*, N=51	*A. hydrophila/caviae*	45	88.2%
	*A. hydrophila*	3	5.9%
	*Aeromonas* spp.	2	3.9%
	*A. sobria*	1	2.0%
*A. caviae*, N=55	*A. hydrophila/caviae*	48	87.3%
	*A. caviae*	4	7.3%
	*Aeromonas* spp.	2	3.6%
	*A. hydrophila*	1	1.8%
*A. veronii*, N=69	*A. sobria*	32	46.4%
	*Aeromonas* spp.	27	39.1%
	*A. hydrophila/caviae*	7	10.1%
	*A. veronii*	2	2.9%
	*A. caviae*	1	1.4%
*A. sobria*, N=7	*A. sobria*	5	71.4%
	*A. veronii*	1	14.3%
	*Aeromonas* spp.	1	14.3%
*A. enteropelogenes*, N=1	*Aeromonas* spp.	1	100.0%
*A. sanarellii*, N=2	*A. hydrophila/caviae*	2	100.0%

### The Difference in Clinical Characteristics, Virulence Genes, Resistance Genes, and Antimicrobial Susceptibility Between Species

Two patients had two different *Aeromonas* species isolated from the same specimen, and another two had two different *Aeromonas* species isolated from two consecutive blood cultures. All were omitted in the following analysis. *A. veronii* was the most common species isolated in bacteremic patients (28/69, 40.6%), followed by *A. caviae* (19/69, 27.5%), *A. hydrophila* (13/69, 18.8%), and *A. dhakensis* (6/69, 8.7%). *A. hydrophila* (16/36, 44.4%) and *A. dhakensis* (12/36, 33.3%) predominated in the skin and soft tissue infection (p<0.0001), whereas *A. veronii* (13/29, 44.8%) and *A. caviae* (10/29, 34.5%) prevailed in biliary tract infections (p=0.011). *A. caviae* preponderates in pneumonia (8/13, 61.5%, p=0.017), while *A. dhakensis* (7/22, 31.8%) and *A. caviae* (6/22, 27.3%) dominated in intra-abdominal infections (p=0.012). Patients with hematological malignancies were infected by exclusively *A. veronii* and *A. hydrophila* (8/13, 61.5% and 5/13, 38.5%, respectively, p=0.014).

As for virulence genes, which were shown in **Table 3**, both *A. dhakensis* and *A. hydrophila* almost invariably carried *col*, *ela*, *fla*, *hlyA*, *lip*, and *alt* (88.2 to 100%). The difference between them was that *A. dhakensis* carried *act* and *aexT* more often and possessed *ast* less frequently without reaching statistical significance. *A. veronii* and *A. sobria* both possessed *act* and *ascV* more often (p<0.0001). *ascF*-*ascG* was found in *A. hydrophila* (31.4%), *A. dhakensis* (24.3%), and *A. veronii* (24.6%).

**Table 3:** Resistance and virulence genes among different *Aeromonas* species.

Genes	*A. dhakensis* (37)	*A. hydrophila* (51)	*A. caviae* (55)	*A. veronii* (69)	*A. sobria* (7)	Others (3)	Total (222)
**Resistance**							
*bla*_TEM_	2 (5.4)	0 (0.0)	2 (3.6)	0 (0.0)	0 (0.0)	0 (0.0)	4 (1.8)
*bla*_SHV_	0 (0.0)	0 (0.0)	3 (5.5)	0 (0.0)	0 (0.0)	0 (0.0)	3 (1.4)
*bla*_PER_	0 (0.0)	0 (0.0)	3 (5.5)	0 (0.0)	0 (0.0)	0 (0.0)	3 (1.4)
*bla_AQU-1_*	34 (91.9)	0 (0.0)	0 (0.0)	0 (0.0)	0 (0.0)	0 (0.0)	34 (15.3)
*bla*_MOX_	0 (0.0)	0 (0.0)	55 (100.0)	0 (0.0)	0 (0.0)	1 (33.3)	56 (25.2)
*bla*_CphA_	37 (100.0)	51 (100.0)	0 (0.0)	62 (89.9)	0 (0.0)	0 (0.0)	150 (67.6)
*bla*_KPC_	0 (0.0)	0 (0.0)	0 (0.0)	0 (0.0)	0 (0.0)	0 (0.0)	0 (0.0)
*bla*_IMP_	0 (0.0)	0 (0.0)	0 (0.0)	0 (0.0)	0 (0.0)	0 (0.0)	0 (0.0)
*bla*_VIM_	0 (0.0)	0 (0.0)	0 (0.0)	0 (0.0)	0 (0.0)	0 (0.0)	0 (0.0)
*bla*_NDM_	0 (0.0)	0 (0.0)	5 (9.1)	0 (0.0)	0 (0.0)	0 (0.0)	5 (2.3)
*bla*_OXA-48-like_	0 (0.0)	0 (0.0)	0 (0.0)	0 (0.0)	0 (0.0)	0 (0.0)	0 (0.0)
*bla*_OXA-23-like_	0 (0.0)	0 (0.0)	0 (0.0)	0 (0.0)	0 (0.0)	0 (0.0)	0 (0.0)
**Virulence**							
*aexT*	4 (10.8)	0 (0.0)	0 (0.0)	15 (21.7)	0 (0.0)	0 (0.0)	19 (8.6)
*act*	17 (45.9)	10 (19.6)	1 (1.8)	46 (66.7)	6 (85.7)	0 (0.0)	80 (36.0)
*aerA*	8 (21.6)	7 (13.7)	0 (0.0)	0 (0.0)	0 (0.0)	0 (0.0)	15 (6.8)
*Alt*	36 (97.3)	45 (88.2)	3 (5.5)	11 (15.9)	1 (14.3)	0 (0.0)	96 (43.2)
*ascF-ascG*	9 (24.3)	16 (31.4)	0 (0.0)	17 (24.6)	0 (0.0)	0 (0.0)	42 (18.9)
*ascV*	5 (13.5)	7 (13.7)	0 (0.0)	26 (37.7)	5 (71.4)	0 (0.0)	43 (19.4)
*ast*	2 (5.4)	13 (25.5)	1 (1.8)	0 (0.0)	0 (0.0)	0 (0.0)	16 (7.2)
*col*	37 (100.0)	50 (98.0)	24 (43.6)	3 (4.3)	0 (0.0)	2 (66.7)	116 (52.3)
*ela*	37 (100.0)	51 (100.0)	33 (60.0)	4 (5.8)	0 (0.0)	2 (66.7)	127 (57.2)
*fla*	35 (94.6)	51 (100.0)	8 (14.5)	24 (34.8)	5 (71.4)	0 (0.0)	123 (55.4)
*hlyA*	37 (100.0)	51 (100.0)	0 (0.0)	0 (0.0)	0 (0.0)	0 (0.0)	88 (39.6)
*lip*	37 (100.0)	51 (100.0)	33 (60.0)	1 (1.4)	0 (0.0)	2 (66.7)	124 (55.9)

Regarding resistance genes, the ﻿AmpC β-lactamase gene *bla_AQU-1_* was exclusive for *A. dhakensis* isolates (34/37, 91.9%), and *bla_MOX_* was ﻿present in all *A. caviae* isolates. The ﻿metallo-β-lactamase (MBL) gene *bla_CphA_* was ﻿present in ﻿all *A. dhakensis* and *A. hydrophila* isolates and most of the *A. veronii* (62/69, 81.6%) isolates, but not in *A. caviae* or *A. sobria*. 9.1% (5/55) of *A. caviae* isolates carried New Delhi Metallo-beta-lactamase (*bla_NDM_*). 14.5% (8/55) of *A. caviae* and 10.8% (4/37) of *A. dhakensis* isolates also carried extended-spectrum β-lactamase (ESBL) genes (ex. *bla_TEM_*, *bla_SHV_*, *bla_PER_*, and *bla_CTX-M_*_)_. None of the 222 isolates possess other metallo-β-lactamases such as *bla_KPC_*, *bla_IMP_*, *bla_VIM_*, *bla_OXA-23-like_*, and *bla_OXA-48-like_*.

The antimicrobial susceptibility test was conducted for 220 isolates, and the results were shown in **Table 4**. Both *A. dhakensis* and *A. hydrophila* showed reduced susceptibility to cefotaxime and ertapenem (81.1% and 88.2% for cefotaxime and 73% and 84.3% for ertapenem, respectively). The ertapenem susceptibility was decreased in *A. veronii* and *A. sobria* (66.7% and 42.9%, respectively). *A. caviae* was less susceptible to cefotaxime (67.3%). *A. sanarellii* carried *bla_MOX_* and displayed 100% resistance to third-generation cephalosporins such as cefotaxime and ceftazidime, as well as reduced susceptibility to ertapenem (50%). Nearly 90% of *Aeromonas* isolates were susceptible to cefepime, tigecycline, and levofloxacin.

**Table 4:** Antimicrobial susceptibility results of *Aeromonas* clinical isolates.

Antibiotics	No. (%) of Isolates						
	*A. dhakensis*	*A. hydrophila*	*A. caviae*	*A. veronii*	*A. sobria*	Others	Total
**SAM**	3 (8.1)	4 (7.8)	10 (18.2)	12 (17.4)	0 (0)	1 (33.3)	30 (13.5)
**TZP**	27 (73.0)	47 (92.2)	45 (81.8)	56 (81.2)	6 (85.7)	1 (33.3)	182 (82.0)
**CTX**	30 (81.1)	45 (88.2)	37 (67.3)	69 (100)	6 (85.7)	1 (33.3)	188 (84.7)
**CAZ**	31 (83.8)	46 (90.2)	41 (74.5)	69 (100)	6 (85.7)	1 (33.3)	194 (87.4)
**FEP**	35 (94.6)	50 (98.0)	48 (87.3)	69 (100)	7 (100)	3 (100)	212 (95.5)
**ETP**	27 (73.0)	43 (84.3%)	52 (94.5%)	46 (66.7%)	3 (42.9%)	2 (66.6)	173 (77.9%)
**IPM**	32 (86.5%)	49 (96.1%)	53 (96.4%)	65 (94.2%)	7 (100%)	3 (100)	209 (94.1%)
**LVX**	37 (100)	51 (100)	53 (96.4)	69 (100)	7 (100)	3 (100)	220 (99.1)
**GM**	37 (100)	50 (98.0)	52 (94.5)	69 (100)	7 (100)	3 (100)	218 (98.2)
**SXT**	36 (97.3)	46 (90.2)	35 (63.6)	62 (89.9)	6 (85.7)	3 (100)	188 (84.7)
**TGC**	37 (100)	49 (96.1)	54 (98.2)	68 (98.6)	7 (100)	3 (100)	218 (98.2)

SAM, ampicillin/sulbactam; TZP, piperacillin/tazobactam; CTX, cefotaxime; CAZ, ceftazidime; FEP, cefepime; ETP, ertapenem; IMP, imipenem-cilastatin; LVX, levofloxacin; GM, gentamicin; SXT, co-trimoxazole; TGC, tigecycline.

### Risk Factors for Mortality

As shown in **Table 5**, non-survivors were older (p=0.030), tended to have pneumonia (p < 0.0001), and malignancy (p=0.002) when compared with non-survivors in univariate analysis. In univariate analysis, non-survivors were older (p=0.030), tended to have pneumonia (p<0.0001), and malignancy (p=0.002) when compared with non-survivors. There was no significant difference in mortality between different species or cancer types. In multivariate logistic regression analysis, independent risk factors associated with mortality were pneumonia (adjusted odds ratio (aOR)=32.0, p<0.0001), malignancy (aOR=6.6, p=0.001), and *ascF*-*ascG* carriage (aOR=3.5, p=0.026).

**Table 5:** Univariate and multivariate analysis pf risk factors for patients with *Aeromonas* infection.

Factors	Survivors (N = 135) No. (%)	Non-survivors (N = 28) No. (%)	Univariate	Multivariate		
			P value	aOR	95% CI	P value
Age	61.8 ± 17.3	69.3 ± 11.7	0.030			>0.05
Sex, female	47 (34.8)	7 (25)	0.32			
Species, *A. hydrophila*	27 (19.1)	9 (32.1)	0.16	2.6	0.91-7.65	0.08
Polymicrobial Infection	79 (58.5)	16 (57.1)	0.89			
Bacteremia	57 (42.2)	14 (50.0)	0.45			
**Virulence Genes**						
*ascF-ascG*	21 (15.6)	8 (28.6)	0.11	3.5	1.16-10.47	0.026
*ascV*	23 (17.0)	8 (28.6)	0.16			
*aerA*	6 (4.4)	3 (10.7)	0.19			
**Infection Site**						
Pneumonia	4 (3.0)	8 (28.6)	<0.0001	32.0	6.46-158.28	<0.0001
SSTI	35 (25.9)	3 (10.7)	0.08	NS		>0.05
BTI	26 (19.3)	2 (7.1)	0.17	NS		>0.05
**Underlying Disease**						
Biliary stone	15 (11.2)	2 (7.1)	0.74			
Liver cirrhosis/chronic hepatitis	33 (23.1)	10 (32.1)	0.22			
DM	41 (30.4)	4 (14.3)	0.08	NS		>0.05
CVA	5 (3.7)	3 (10.7)	0.14	NS		>0.05
CKD (including H/D)	21 (15.6)	8 (28.6)	0.11	NS		>0.05
Autoimmune disease	2 (1.5)	0 (0)	1.00			
Active malignancy	54 (40.0)	20 (71.4)	0.002	6.6	2.18-19.89	0.001

SSTI, skin and soft tissue infection; H/D, hemodialysis; aOR, adjusted odds ratio; NS: not statistically significant.

## Discussion

The prevalence of human infections caused by *A. veronii* and *A. dhakensis* might be underestimated since both would be misidentified as *A. hydrophila* or *A. sobria* by the phenotype-based identification system or even MALDI-TOF MS as shown in the present study. ﻿*A. dhakensis* was found to be the most common *Aeromonas* species isolated from wound cultures, more virulent than *A. hydrophila ex vivo* and in animal models (Chen et al., 2014), as well as harboring the highest 14-day sepsis-related mortality rate among monomicrobial *Aeromonas* bacteremia (Wu et al., 2015). *A. dhakensis* was found to be the dominant aeromonad in Singapore and Malaysia, accounting for 45-50% of all *Aeromonas* species identified (Puthucheary et al., 2012; Khor et al., 2018). In Australia, *A. dhakensis* was the most prevalent aeromonad in clinical and water samples, especially in wounds (Aravena-Román et al., 2011). T3SSs are found in many Gram-negative bacterial pathogens including *Pseudomonas*, *Yersinia*, *Salmonella, Shigella*, as well as enteropathogenic and enterohemorrhagic *Escherichia coli* (Wu et al., 2007). The T3SS of *Aeromonas* is similar to that of *Yersinia* (Vilches et al., 2004), with at least 21 effector proteins (Rangel et al., 2019) that exhibit cytotoxicity, induce apoptosis, reduce phagocytosis, and trigger cytokines/chemokines production (Yu et al., 2004; Burr et al., 2005; Sierra et al., 2010). Strains of *A. salmonicida* and *A. hydrophila* with mutations in the T3SS apparatus were shown to be less virulent than non-mutated strains (Vilches et al., 2004; Yu et al., 2004; Burr et al., 2005). Our previous research demonstrated that *ascF-ascG* was mainly present in *A. hydrophila*, *A. dhakensis*, and *A. veronii* (50%, 14.3%, and 1%, respectively) (Wu et al., 2019), and *ascV* was previously more common in *A. hydrophila* comparing with *A. dhakensis* (92.3% vs 51.4%, p=0.017) (Chen et al., 2014), but there were shreds of evidence demonstrating an association between the presence of *ascV*, *aexT* or *ascF-ascG* genes and the development of extraintestinal infections or bacteremia among patients with *Aeromonas* isolates (Wu et al., 2007). The present study illustrated that *ascV* carriage was similar between *A. hydrophila* and *A. dhakensis* (13.7% vs 13.5%), and *A. veronii* possessed *ascF-ascG* gene more often than previously reported. The *ascF-ascG* gene was independently correlated to crude in-hospital mortality in the present study, a correlation that had not yet been elucidated in other studies.

The distribution of the AmpC β-lactamases and MBL genes were found to be species-specific in a previous study conducted in our hospital, with all *A. dhakensis*, *A. caviae*, and *A. hydrophila* isolates carrying *bla_AQU-1_*, *bla_MOX_*, and *bla_CepH_*, respectively (Wu et al., 2015). Consistent with this finding, the present study demonstrated increased resistance to third-generation cephalosporins among the three aeromonads harboring genes encoding AmpC β-lactamases. In the present study, *bla_AQU-1_* was found exclusively but not universally in 91.9% of the *A. dhakensis* isolates. Reduced susceptibility to cefepime was found among ESBL genes-carrying *A. caviae* and *A. dhakensis* isolates, but 2% of *A. hydrophila* isolates also exhibited cefepime resistance without identifiable ESBL genes in the present study. Resistance to ertapenem was quite high among aeromonads carrying the MBL gene *bla_CphA_*, such as *A. dhakensis*, *A. hydrophila*, and *A. veronii*, and resistance to imipenem could be found in the aforementioned aeromonads, as well as *bla_NDM_*-carrying *A. caviae*. *A. caviae* was found to carry *bla_NDM_* on the chromosome from water seepage samples in New Dehli in 2010 (Walsh et al., 2011). Clinicians should be aware of the emergence of *bla_NDM_* in *A. caviae*. Moreover, 57.1% of *A. sobria* isolates showed intermediate susceptibility to ertapenem without carrying *bla_CphA_* or other carbapenemases tested in the present study. Other carbapenemases, such as the class D penicillinase AmpS, had been discovered in *A. sobria* (Walsh et al., 1995a; Walsh et al., 1995b). The two *A. sanarellii* isolates, one of them carrying *bla_MOX_* gene, displayed non-susceptibility to cefotaxime and piperacillin/tazobactam, and one of them was resistant to ertapenem and tetracycline. Other AmpC β-lactamases and MBL or porin alterations not examined in this study may contribute to the drug resistance.

*Aeromonas* infection had been linked to patients with liver cirrhosis or cancer with poorer outcomes in Taiwan (Ko and Chuang, 1995; Ko et al., 2000; Wang et al., 2009), an island that had been endemic with hepatitis B (Chan et al., 2004). The present study demonstrated that the proportion of patients with active malignancy had surpassed liver cirrhosis as the most common underlying disease in patients with *Aeromonas* infection, possibly attributed to the mass vaccination program of hepatitis B vaccine since 1984, thereby reducing the carrier rate by 85% (Chan et al., 2004). Hepatocellular carcinoma (HCC), which was linked to liver cirrhosis and chronic hepatitis B and C infection, was the most common cancer type in the present study, accounting for 19.4% of all patients with active malignancies, and had the highest crude in-hospital mortality rate (6/14, 42.9%) among all comorbidities. Liver cirrhosis confers susceptibility to infection by immune dysfunction including reduced secretory IgA and bile acid, and alteration in the gut microbiome, making the host susceptible to infections originating from the gut (Bajaj et al., 2021). On the other hand, chemotherapies directed against malignancies confer susceptibility to food-borne infections by disrupting the gut mucosal barrier, and the following neutropenia predisposes the host to opportunistic infections (National Comprehensive Cancer Network, 2020). The risk of infection in patients with HCC and pancreatobiliary cancers may also be increased due to hepatobiliary obstruction caused by tumors, as a sequela of hepatobiliary reconstruction surgery (National Comprehensive Cancer Network, 2020), and resistance to bile salts of aeromonads (Want and Millership, 1990). Chao et al. discovered that patients with cancer are associated with higher mortality in *Aeromonas* bacteremia, pneumonia, and biliary tract infection (Chao et al., 2013a; Chao et al., 2013b; Tang et al., 2014). In contrast, the outcome of skin and soft tissue infection attributed to *Aeromonas*﻿ was associated with diabetes mellitus but not immune status (Chao et al., 2013c).

In the present study, patients with hematologic malignancies were infected by *A. veronii* and *A. hydrophila* exclusively, and the crude in-hospital mortality was 30.8% (4/13). In another tertiary medical center in Southern Taiwan, Tsai et al. found that 35.6% of 41 patients with hematologic dyscrasias succumbed to *Aeromonas* bacteremia within 14 days of onset, with a remarkably high resistant rate to imipenem (35.6%) (Tsai et al., 2006). Patients with hematological malignancies were particularly vulnerable to opportunistic infection owing to the frequent leukopenia due to marrow infiltration of malignant cells or dysfunctional marrow, the severe mucosal damage, and prolonged neutropenia following higher-intensity chemotherapies, and a shift in enteric microbial flora accompanied by severe illness and antimicrobial usage (National Comprehensive Cancer Network, 2020).

Biliary tract infection with aeromonads was associated with biliary stone and pancreatobiliary cancer in our study, agreeing with other studies in southern Taiwan and Japan (Chao et al., 2013a; Kitagawa et al., 2020). Pancreatic cancer was the second most common cancer in our study, and the sum of patients with pancreatobiliary cancers outnumbered patients with HCC. In Japan, pancreatobiliary cancer, liver cirrhosis, and obstructive biliary disease contributed equally to comorbidity in patients with *Aeromonas* bacteremia, and 57.9% of bacteremia originated from biliary tract infection (Kitagawa et al., 2020). The incidence of pancreatobiliary cancers was around 20.93 per 100,000 inhabitants per year in Taiwan, far way behind the incidence of breast cancer, colorectal cancer, lung cancer, prostate cancer, and hepatocellular carcinoma (119.71, 70.05, 65.05, 56.72, and 36.62 per 100,000 inhabitants per year, respectively) (Health Promotion Administration, Ministry of Health and Welfare, 2020). This phenomenon may be attributed to the preponderance and even possible carcinogenesis of aeromonads in patients with pancreatobiliary cancers, or simply an institutional bias since our hospital was renowned for the treatment of pancreatic cancer (Su et al., 2020). Overexpression of the p38 MAPK pathway is observed in the pancreatic cancer cells and hepatocellular cells (Dhillon et al., 2007). Our previous research (Chen et al., 2018) have discovered that A. dhakensis infection causes p38 mitogen-activated protein kinase (MAPK) pathway activation in Caenorhabditis elegans model. We presume that Aeromonas species living in the hepatobiliary tract trigger the development of cancers by activating p38 MAPK pathway. Further studies are warranted to clarify the causality between *Aeromonas* and pancreatobiliary cancer. Half (8/16) of the patients with biliary stones were infected with *A. caviae* and half (6/12) of those with pancreatic cancer with *A. veronii*. Since *A. caviae* and *A. veronii* carry *bla_MOX_* and *bla_CphA_*, respectively, cefepime and fluoroquinolone are drugs of choice in these patients.

Three-fourths of the patients with *Aeromonas* pneumonia died in the hospital. Pneumonia was the most significant factor associated with crude in-hospital mortality in the present study, yet it was caused by *A. caviae*, the least virulent species with the highest resistance to third-generation cephalosporins and therefore most healthcare-associated among the commonly encountered aeromonads (Wu et al., 2015). A study conducted in another hospital located in the same city addressing 84 patients with *Aeromonas* pneumonia showed that the in-hospital mortality was merely 28.6% (Chao et al., 2013c). Since the majority (10/13, 76.9%) of these patients had a polymicrobial infection, the high mortality in the present may reflect the complex comorbidities and prolonged hospitalization, not the virulence of *Aeromonas* itself.

This study had several limitations. First, clinical information was collected retrospectively. Selection bias may be present since there could be more severe patients and more patients with rarer malignancies such as pancreatobiliary carcinomas were referred to the study hospital. Second, this study was conducted in a single medical center, and a multicenter study is warranted for a more comprehensive understanding of the epidemiology of clinical infections caused by aeromonads in other areas. Finally, a severity score was not available in this study, which may contribute to the risk of mortality in the outcome analysis.

## Conclusion

*A. veronii*, *A. caviae*, *A. hydrophila*, and *A. dhakensis* were the most frequently isolated species in *Aeromonas* infections. Infection with *ascF-ascG Aeromonas* and underlying malignancies were associated with mortality. Cefepime, fluoroquinolones, and tigecycline are the drugs of choice for *Aeromonas* infections, especially for skin and soft tissue infections and biliary tract infections in patients with underlying pancreaticobiliary cancers.

## Authors Contributions

P-LC conceived and designed the experiments. S-LS performed all the experiments. Y-WC analyzed the data and drafted the paper. C-WL, N-YL, and C-ST provided technical help on data analysis. C-CL, W-CK, C-LL, L-SS, and M-CL critically commented on the analysis. P-LC reviewed and edited the paper. All authors contributed to the article and approved the submitted version.

## Data Availability Statement

The original contributions presented in the study are included in the article/Supplementary Material. Further inquiries can be directed to the corresponding author.

## Funding

The study was partially supported by research grants from the Ministry of Science and Technology, Taiwan (MOST 110-2628-B-006-028), and National Cheng Kung University Hospital (NCKUH-10902001 and NCKUH-110040027).

## Conflict of Interest

The authors declare that the research was conducted in the absence of any commercial or financial relationships that could be construed as a potential conflict of interest.

## Publisher’s Note

All claims expressed in this article are solely those of the authors and do not necessarily represent those of their affiliated organizations, or those of the publisher, the editors and the reviewers. Any product that may be evaluated in this article, or claim that may be made by its manufacturer, is not guaranteed or endorsed by the publisher.
